# Group Music Intervention for Persons With Dementia in Assisted Living: A Pilot Study

**DOI:** 10.1093/geroni/igaa057.3088

**Published:** 2020-12-16

**Authors:** Hongdao Meng, Jennifer Bugos, Debra Dobbs, Soomi Lee, Punam Risal, Britney Veal, William Patterson

**Affiliations:** 1 University of South Florida, Tampa, Florida, United States; 2 University of South Florida, Winter Haven, Florida, United States; 3 Digital Media Innovations and eLearning Enterprises, Tampa, Florida, United States

## Abstract

Dementia is the third leading diagnosis among US residents in assisted living communities (ALCs), and agitation is a major challenge for residents, families, and staff. While music interventions in nursing homes and the community have generated promising findings, little evidence of acceptability or efficacy data are available in ALCs. This pilot study tested the acceptability and preliminary efficacy of a staff-led group music intervention among ALC residents with dementia (n=19). We used a mixed-methods pre-post study design. The primary outcome measure was the Cohen-Mansfield Agitation Inventory-Short Form (CMAI-SF), and facilitator focus group interviews were conducted to gain additional insight into intervention acceptability and facilitators/barriers to implementation among activity staff. Seventeen (89.5%) participants completed the intervention with a 77% overall session attendance rate. Results suggest that the intervention is well-received by management, family, and activity staff, Implications for intervention design, efficacy testing, and contextual factors related to implementation will be discussed.

